# Characterization and Correction of Bias Due to Nonparticipation and the Degree of Loyalty in Large-Scale Finnish Loyalty Card Data on Grocery Purchases: Cohort Study

**DOI:** 10.2196/18059

**Published:** 2020-07-15

**Authors:** Anna-Leena Vuorinen, Maijaliisa Erkkola, Mikael Fogelholm, Satu Kinnunen, Hannu Saarijärvi, Liisa Uusitalo, Turkka Näppilä, Jaakko Nevalainen

**Affiliations:** 1 Faculty of Social Sciences (Health Sciences) Tampere University Tampere Finland; 2 VTT Technical Research Centre of Finland Ltd Tampere Finland; 3 Department of Food and Nutrition University of Helsinki Helsinki Finland; 4 Faculty of Management and Business Tampere University Tampere Finland; 5 Tampere University Library Tampere University Tampere Finland

**Keywords:** loyalty card data, diet, selection bias, weighting, raking, food

## Abstract

**Background:**

To date, the evaluation of diet has mostly been based on questionnaires and diaries that have their limitations in terms of being time and resource intensive, and a tendency toward social desirability. Loyalty card data obtained in retailing provides timely and objective information on diet-related behaviors. In Finland, the market is highly concentrated, which provides a unique opportunity to investigate diet through grocery purchases.

**Objective:**

The aims of this study were as follows: (1) to investigate and quantify the selection bias in large-scale (n=47,066) loyalty card (LoCard) data and correct the bias by developing weighting schemes and (2) to investigate how the degree of loyalty relates to food purchases.

**Methods:**

Members of a loyalty card program from a large retailer in Finland were contacted via email and invited to take part in the study, which involved consenting to the release of their grocery purchase data for research purposes. Participants’ sociodemographic background was obtained through a web-based questionnaire and was compared to that of the general Finnish adult population obtained via Statistics Finland. To match the distributions of sociodemographic variables, poststratification weights were constructed by using the raking method. The degree of loyalty was self-estimated on a 5-point rating scale.

**Results:**

On comparing our study sample with the general Finnish adult population, in our sample, there were more women (65.25%, 30,696/47,045 vs 51.12%, 2,273,139/4,446,869), individuals with higher education (56.91%, 20,684/36,348 vs 32.21%, 1,432,276/4,446,869), and employed individuals (60.53%, 22,086/36,487 vs 52.35%, 2,327,730/4,446,869). Additionally, in our sample, there was underrepresentation of individuals aged under 30 years (14.44%, 6,791/47,045 vs 18.04%, 802,295/4,446,869) and over 70 years (7.94%, 3,735/47,045 vs 18.20%, 809,317/4,446,869), as well as retired individuals (23.51%, 8,578/36,487 vs 31.82%, 1,414,785/4,446,869). Food purchases differed by the degree of loyalty, with higher shares of vegetable, red meat & processed meat, and fat spread purchases in the higher loyalty groups.

**Conclusions:**

Individuals who consented to the use of their loyalty card data for research purposes tended to diverge from the general Finnish adult population. However, the high volume of data enabled the inclusion of sociodemographically diverse subgroups and successful correction of the differences found in the distributions of sociodemographic variables. In addition, it seems that food purchases differ according to the degree of loyalty, which should be taken into account when researching loyalty card data. Despite the limitations, loyalty card data provide a cost-effective approach to reach large groups of people, including hard-to-reach population subgroups.

## Introduction

Diet has a substantial impact on human health. Poor dietary habits are associated with obesity and a wide range of chronic diseases, including type 2 diabetes, cancer, and cardiovascular diseases [1,2]. Suboptimal diet is responsible for more deaths than any other risk factor globally [3]. It is therefore imperative to collect timely and valid information on diet and individual risk factors.

To date, the evaluation of diet has mostly been based on questionnaires and diaries [4]. Although valuable in research, data collection with such instruments, particularly food diaries, is time and resource intensive, and the information is gained with a considerable delay. They also suffer from participant tendency toward social desirability [5,6]. Moreover, the information gained through questionnaires is subject to recall bias with participants not reporting all foods consumed [4]. Another limitation with dietary surveys as well as health surveys in general is selection bias, which manifests as healthy, socioeconomically advantaged, middle-aged women being the most likely to enroll in these studies [7-9].

The continued development of innovative digital tools and digital data repositories provides novel opportunities for epidemiological research [10-13]. Web-based data collection instruments [13,14] and consumer-generated data are increasingly being used for health research purposes [15-19]. While such novel data collection methods and tools may overcome some of the problems faced with traditional methods, some of the limitations remain, of which selection bias is a major concern [13,20]. Namely, those who generate the data are frequently highly selected and likely to differ from the general population representing wealthy and healthy individuals. For instance, smartphone users, and subsequently mobile health app and social media users, are younger, better educated, and represent wealthier individuals than those in the general population [21-23]. However, automated data collection, which is a typical feature for these instruments and tools, provides objective measures on individuals’ health behaviors and thus decreases information bias.

Food purchase data have invoked interest as a novel approach to enrich diet and nutrition research efforts [24-26]. So far, most of the published studies have used panel-based data, with all grocery purchase receipts scanned at home [26]. While such studies are frequently large and may include data from multiple sources, they are limited by recording discrepancies [27]. In addition, receipt scanning requires consistent efforts and long-term engagement from the participants [28,29]. In this study, we used loyalty card data (ie, individual-level grocery transaction data generated by retail food chains). Importantly, loyalty card data contain information about what, where, when, and who has bought, thus enabling longitudinal tracking of the purchase behaviors of a single customer or a household over time. Objective measures of food purchases have been shown to correlate with one’s food intake and overall diet quality [28]. Loyalty card data also accumulates automatically in retailers’ information technology systems, producing objective and up-to-date information in a cost-effective manner. However, loyalty card data have shortfalls that could impede the usefulness for research. First, consumers may distribute their purchases among different retailers. Therefore, loyalty card data from a single retailer most likely does not include all food purchases conducted by consumers. However, in Finland, the market is highly centralized with the three biggest market chains claiming over 90% of the market share, and the largest operator having a market share as high as 47% [30]. Such centralization provides a unique opportunity to investigate heterogeneous populations through a single retailer.

The aims of this study were as follows: (1) to investigate and quantify selection bias in Finnish large-scale loyalty card (LoCard) data and further develop a means to correct this bias by characterizing the loyalty card data consenters and comparing their sociodemographic background to that of the general Finnish adult population and (2) to assess how the degree of loyalty relates to food purchases by investigating the self-perceived degree of loyalty (share of total grocery purchases in retailers’ shops and supermarkets) and its association with food purchases. The overall purpose of this research was to increase the understanding of how loyalty card data should be understood and subsequently analyzed in dietary and health research.

## Methods

### Study Design and Participation

The LoCard data used in this study were obtained from S Group, which is the largest commercial operator of retail grocery stores in Finland. According to S Group, their full coverage is 2.4 million households, meaning that 88% of households in Finland have registered purchases in their databases. The members of S Group’s loyalty card program are provided with an electronic customer card to be used when making purchases, and customers are rewarded for their purchases by getting a maximum 5% financial bonus that is refunded to them on a monthly basis. Individuals of the same household may link their purchases to the same loyalty account. In this study, only purchases of the household’s main cardholder were used.

Members of S Group’s loyalty card program (primary cardholders) across Finland were contacted via email and were invited to take part in the study, which involved consenting to the release of their grocery purchase data to be used for research purposes and voluntarily responding to the study questionnaire. Members who did not have an email address declared or who had prohibited the retailer from contact them with any marketing or research-related material were excluded. Cardholders under 18 years of age were also excluded. All invitations were sent by S Group as they had customers’ contact information.

The grocery purchase data used in this study covered the period from January 1, 2017, to December 31, 2018. Each purchase was associated with item description, time stamp, quantity (ie, weight, volume, or number of packages), and expenditure on the item.

### Background Variables

All consenting participants were asked to fill out a web-based background questionnaire that included the following sociodemographic variables: education, marital status, size of the household, number and age of children, occupational status, income, and perceived health. The background data were complemented with information on participant sex, age, and postal code obtained from the retailer’s electronic database.

### Degree of Loyalty

As part of the baseline questionnaire, all participants were asked to estimate their degree of loyalty as a share of purchases made in the retailer’s shops and supermarkets on a five-item ordinal scale. The response categories were as follows: “0%-20%,” “21%-40%,” “41%-60%,” “61%-80%,” and “81%-100%.”

### Food Variables and Food Groups

The LoCard grocery purchase data required preprocessing to be usable in further analyses. First, we identified food groups from all the grocery product groups. Second, we regrouped the identified food groups into new groups that were formed on the basis of the commonly used food groupings in nutritional studies [31] and earlier findings on the associations between dietary components and health [32,33]. For instance, skimmed liquid milk and buttermilk were aggregated into “skimmed milk & sour milk” and foods and mixed dishes with red or processed meat as the main ingredient were aggregated into “red meat & processed meat.”

Out of 4234 grocery product groups, 865 (20.4%) were assigned into one of the new food groups used in this study. In addition, 42 food groups were left out as they involved either (1) a mixed dish or food group with no definite primary ingredient or (2) a rarely purchased product. The food groups used in this study included “vegetables,” “skimmed milk & sour milk,” “sugar-sweetened beverages,” “rye bread,” “red meat & processed meat,” “fat spreads,” and “sweets & chocolate.” These groups were used as indicators for evaluating the nutritional quality of household food purchases. A detailed description about the grouping of the food purchase data is included in Multimedia Appendix 1.

### Reference Material

Population statistics on the general adult population were obtained from Statistics Finland using StatFin databases that can be freely accessed [34]. The databases include tabulated data on Finnish citizens and Finland in general that are collected on a yearly basis. Data from 2017 were used because of the availability of the latest data tables for all sociodemographic variables used in the analyses. For this study, individuals aged at least 18 years were included.

The FinHealth survey is a national population health study on Finnish citizens. The study encompasses a series of cross-sectional population surveys carried out every 5 years in Finland. The latest FinHealth survey was carried out at 50 localities in 2017, with a participation rate of 71% among those invited for the study [35]. The purpose of the FinHealth study is to collect up-to-date information about the health and well-being of adults residing in Finland and on the factors influencing their health and well-being. Each survey invites 10,000 randomly selected individuals aged over 18 years. The study consists of physical examinations and study questionnaires. The latest report (values used in this study) is restricted to adults aged 30 years or older to make the results comparable with earlier FinHealth studies. A subgroup of the participants was also invited to undergo a nutrition review; the FinDiet survey is a substudy (n=1655) of the FinHealth survey, which monitors the nutrition and dietary habits of the Finnish population [36].

### Statistical Methods

#### Analysis of and Correction for Selection Bias

The sociodemographic characteristics of the LoCard study participants were first compared with the characteristics of the Finnish adult population and participants of the FinHealth study to identify traits in LoCard participants that deviated from traits in the general Finnish adult population.

Second, we constructed poststratification weights for the LoCard participants to match their sociodemographic distributions with the adult Finnish population distributions as closely as possible. The individual weights were calculated using the raking function available in the *survey* package in R [37]. The raking function uses iterative proportional fitting (IPF), which is a technique that can be used to adjust a distribution reported in one dataset by totals reported in another. For a given two-way contingency table, the IPF proportionally adjusts each row of the sample distribution in the two-way contingency table to have its total equal the reference population row distribution and adjusts each column of the sample distribution to have its total equal the column total in the reference table [38].

The advantage of the raking function is that the algorithm allows multiple two-dimensional (or higher dimensional) tables to be matched simultaneously [37]. For example, instead of matching age, sex, and education univariate distributions separately, we can match all bivariate distributions (ie, age and education, sex and education, and sex and age) simultaneously. The adjustment process is repeated iteratively until the weights converge for each table used in the analysis. The raking function requires that the two contingency tables have the same classes for the row and column variables and no zero values in any of the cells.

The following two-way tables were available for both the LoCard data and the Finnish adult population: sex and age, sex and education, sex and marital status, sex and occupational status, age and education, age and marital status, age and occupational status, and education and occupational status. All tables were subsequently used to construct the poststratification weights. In addition, the distribution of children aged under 18 years living in the household was used alone because corresponding two-dimensional tables with any of the background variables were not available in Statistics Finland. In total, eight two-way tables and a single one-way table were used in the construction of the weights. Finally, the obtained weights were trimmed to avoid extreme values and instability by setting a minimum value of 0.1 and a maximum value of 10. Without trimming, the poststratification weights ranged from 0.04 to 32.7, and there was a single extremely high weight of 82.4.

Owing to missing data, the poststratification weights were constructed in two phases. First, the weights were calculated as described above for participants for whom all baseline characteristics used in the matching were available. These data were available for 36,094 individuals. Participants with missing data for any of these variables (n=10,972) obtained their weights in the second phase, where the poststratification weights were calculated for the whole LoCard sample using sex and age variables only. This information was available for 47,045 participants. Finally, the combined weights were rescaled to add up to 47,045. Twenty-one participants without data on sex and/or age remained without weights.

The selected food group variables were analyzed to describe the volume and money (€) spent on their purchases over the 2-year period (2017-2018). For descriptive purposes, median values and IQRs were reported for each variable because the distributions were strongly skewed to the right, and there was an excess number of zero values in some of the food variables. The same variables were used to demonstrate how the poststratification weights affected the results.

#### Degree of Loyalty

To validate the self-assessed degree of loyalty, we conducted the recency, frequency, and monetary (RFM) value analysis using the transaction data of all participants and compared the RFM scores across the five degree of loyalty groups. RFM analysis is a behavior-based technique used to segment customers by examining their transaction history from three dimensions (how recently a customer made purchases, how often they purchased, and how much they purchased). RFM analysis is also widely used in customer relationship management. Based on these three dimensions, the RFM score is generated for each individual, with a higher score indicating higher loyalty. The analysis was conducted using the *rfm* package in R [39]. In addition, total volume and total money (€) spent on food purchases were calculated for each degree of loyalty group to investigate how closely the self-reported degree of loyalty relates to volume and money spent on the purchases.

To assess the impact of the degree of loyalty on food purchasing profiles, the selected food group variables were compared among the five degree of loyalty groups. The Kruskal-Wallis test was applied for differences across the groups.

The association between the degree of loyalty and background characteristics was analyzed by comparing the distribution of each sociodemographic variable among the five degree of loyalty groups. The differences across the groups were tested using the chi-square test.

### Ethical Aspects

The study was approved by the University of Helsinki Review Board in the Humanities and Social and Behavioral Sciences (Statement 21/2018). Informed consent was electronically obtained from all participants included in the study when they were invited via email to release their loyalty card data and fill out the background questionnaire. The data were pseudonymized by S Group before the researchers could obtain the data.

## Results

### Recruitment

**Figure 1:**
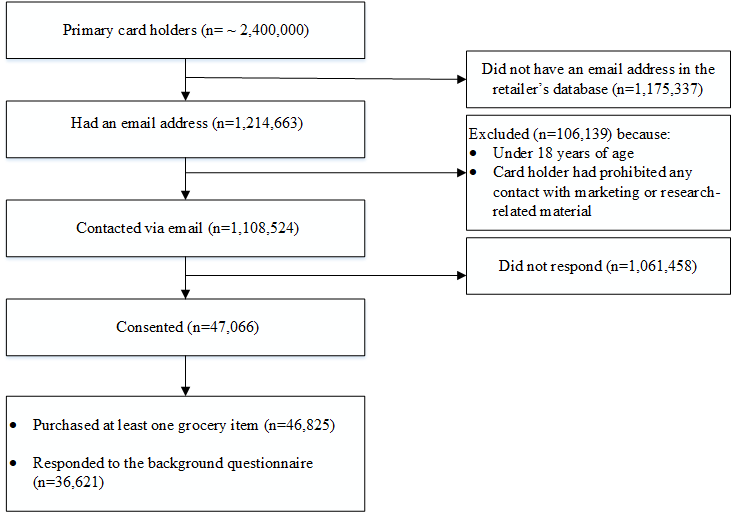
Participant recruitment and eligibility flow chart.

S Group had approximately 2.4 million primary loyalty card owners, and all of them were assessed for eligibility (Figure 1). Approximately half (1,214,663, 51%) of the loyalty card owners were contacted, and of these, 47,066 (4%) consented to participate. We did not have information on the number of valid email addresses or what proportion of emails reached the card owners (eg, by passing through trash email filters). Among the participants, 36,621 (78%) responded to the background questionnaire. Nearly all participants (46,825, 99.5%) purchased at least one grocery item from 2017 to 2018.

### Participant Characteristics

Table 1 shows the participant characteristics compared with those of the Finnish adult population and the FinHealth study participants. Discrepancies were found in sex, age, education, and occupational status when compared with the general Finnish adult population. Namely, there were more women, more individuals with a higher education, and more employed individuals in the LoCard sample. On the contrary, individuals aged under 30 years and over 70 years (correspondingly, retired individuals) were underrepresented in the LoCard sample. Selectivity associated with education was strong in the LoCard sample. The proportion of individuals having a basic education level was clearly lower in the LoCard sample (6% of participants had basic education) than in the Finnish adult population (25% had basic education). There were no major differences in the distribution of marital status. However, there were fewer individuals living in a household with children aged under 18 years in the LoCard sample. The LoCard sample was widely distributed across Finland and comparable to the geographical distribution of Finnish citizens (Multimedia Appendix 2 and Multimedia Appendix 3).

On comparing the LoCard sample to the FinHealth study participants, there were differences in sex, education, and marital status distributions, with more women and individuals with higher education and fewer married individuals in the LoCard sample. The age distributions were not comparable owing to the fact that the FinHealth study included only individuals aged at least 30 years. Distortion in the distribution of occupational status was similar in the two studies compared with the Finnish adult population.

The reweighted distributions of the sociodemographic variables demonstrated that the constructed poststratification weights corrected the deviations successfully, and thereafter, the sociodemographic distributions of the LoCard sample matched well with the Finnish adult population.

**Table 1:** LoCard participant characteristics compared with those of the general Finnish adult population and participants of the FinHealth study.

Characteristic		Finnish general adult population (N=4,446,869)	FinHealth study^a^ (N=6545)	LoCard sample (N=47,066)^b^	Weighted LoCard sample^c^ (N=47,045)
Sex (women), n (%)		2,273,139 (51.12%)	3496 (53.42%)	30,696 (65.25%)	23,837 (50.67%)
Age (years), mean (SD)		50.23 (19.06)	—^d^	47.10 (15.21)	49.5 (0.14)
**Age distribution (years), n (%)**					
	≤29	802,295 (18.04%)	N/A^e^	6791 (14.44%)	8532 (18.14%)
	30-39	702,767 (15.80%)	Men, 483 (15.8%); women, 539 (15.4%)	9982 (21.22%)	7505 (15.95%)
	40-49	660,703 (14.86%)	Men, 530 (17.4%); women, 561 (16.1%)	9503 (20.20%)	6986 (14.85%)
	50-59	734,554 (16.52%)	Men, 608 (20.0%); women, 661 (18.9%)	9154 (19.45%)	7715 (16.40%)
	60-69	737,233 (16.58%)	Men, 727 (23.8%); women, 774 (22.4%)	7880 (16.75%)	7734 (16.44%)
	≥70	809,317 (18.20%)	Men, 701 (23.0%); women, 961 (27.5%)	3735 (7.94%)	8572 (18.22%)
**Marital status, n (%)**					
	Presently married	1,990,928 (44.77%)	Men, 58.0%; women, 52.3%	17,240 (47.32%)	16,254 (45.12%)
	Cohabiting	—	Men, 16.7%; women, 14.4%	7408 (20.33%)	—
	Single	1,599,827 (35.98%)^f^	Men, 13.3%; women, 10.3%	6412 (17.60%)	12,762 (35.43%)^f^
	Divorced or separated	574,620 (12.92%)	Men, 8.7%; women, 12.2%	4331 (11.89%)	4713 (13.08%)
	Widowed	281,494 (6.33%)	Men, 3.4%; women, 10.9%	1040 (2.86%)	2295 (6.37%)
Household, mean number of members (SD)		2.8 (not available)	—	2.36 (1.25)	2.42 (0.01)
Children aged under 18 years living in the household, n (%)		566,242 (38.48%)	31^g^ (31.52%)	11,705 (32.08%)	13,567 (37.61%)
**Education, n (%)**					
	Primary school or less	1,112,261 (25.01%)	Men, 23.2%; women, 21.0%	2259 (6.21%)	7881 (23.54%)
	Upper secondary school	1,902,332 (42.78%)	Men, 38.3%; women, 29.1%	13,405 (36.88%)	15,534 (43.25%)
	Bachelor’s degree or equivalent	955,395 (21.49%)	Men, 38.5%^h^; women, 49.9%^h^	11,787 (32.43%)	8453 (21.94%)
	Master’s degree or higher	476,881 (10.72%)	—	8897 (24.48%)	4049 (11.27%)
**Occupational status, n (%)**					
	Employed	2,327,730 (52.35%)	Men, 65.9%; women, 62.3%	22,086 (60.53%)	19,027 (52.75%)
	Unemployed	296,191 (6.66%)	Men, 8.5%; women, 7.0%	1637 (4.49%)	2417 (6.70%)
	Student	230,489 (5.18%)	Men, 2.4%; women, 3.5%	1824 (5.00%)	1619 (4.49%)
	Retired	1,414,785 (31.82%)	Men, 21.3%; women, 20.0%	8578 (23.51%)	11,600 (32.16%)
	Parental leave		Men, 0.2%; women, 4.2%	1255 (3.44%)	—
	Other	177,674 (4.00%)	Men, 1.8%; women, 3.0%	1107 (3.03%)	1411 (3.91%)^i^
**Degree of loyalty, n (%)**					
	0%-20%	—	—	2283 (6.25%)	2132 (5.90%)
	21%-40%	—	—	4670 (12.79%)	4160 (11.52%)
	41%-60%	—	—	6155 (16.85%)	5828 (16.14%)
	61%-80%	—	—	9224 (25.25%)	8962 (24.82%)
	81%-100%	—	—	14,194 (38.86%)	15,031 (41.62%)

^a^FinHealth study included individuals aged ≥30 years, which makes the age distribution not comparable to other data listed in the table.

^b^Data for the following numbers of participants were missing in the LoCard sample: sex, 21; age, 21; marital status, 10,635; household, 10,689; children aged under 18 years, 10,576; education, 10,718; occupational status, 10,579; degree of loyalty, 10,540.

^c^Weighted LoCard sample refers to the descriptive statistics calculated using the poststratification weights of the LoCard participants.

^d^Not available.

^e^N/A: not applicable.

^f^Cohabitating included in this category.

^g^Households with three or more persons.

^h^Bachelor’s degree or higher.

^i^Parental leave included.

### Food Purchase

Table 2 shows the purchases of selected food groups in the original LoCard sample and in the weighted LoCard sample. Over 95% of the participants had purchased at least one food product in all food groups, except skimmed milk & sour milk. Skimmed milk & sour milk had been purchased by 74% of the participants. Among them, the median expenditure and the median weight were €23.0 (€1=US $1.13 in 2017) and 23.5 kg, respectively, during the 2-year follow-up.

After applying the poststratification weights, there was an increase in the purchase of red meat & processed meat and small increases in sugar-sweetened beverages and fat spreads. The purchase of vegetables and sweets & chocolate decreased as a result of reweighting. The largest change was seen in red meat & processed meat; the weighted amount of purchase increased from €387 to €417 (cost) and from 48 kg to 54 kg (weight), corresponding to relative percentage increases of 7.8% and 12.6%, respectively.

**Table 2:** Purchase of selected food groups (measured in € and kg) in the original LoCard sample and in the weighted LoCard sample.

Food group	Original LoCard sample^a^ (N=47,066)				Weighted LoCard sample^a^ (N=47,045)			
	€, median [IQR]	€ (%)^b^, median [IQR]	kg, median [IQR]	kg (%)^c^, median [IQR]	€, median [IQR]	€ (%)^b^, median [IQR]	kg, median [IQR]	kg (%)^c^, median [IQR]
Vegetables	284.3 [124.9-520.1]	7.7 [5.4-10.5]	76.6 [33.2-144.4]	8.2 [5.4-11.7]	263.7 [107.3-487.6]	7.2 [4.8- 9.9]	73.4 [29.6- 139.6]	7.6 [4.8- 11.0]
Skimmed milk & sour milk	6.9 [0-60.9]	0.2 [0-1.7]	7.0 [0-65.6]	0.9 [0-7.3]	6.6 [0-60.5]	0.2 [0-1.8]	6.5 [0-66.0]	0.9 [0-7.2]
Sugar-sweetened beverages	45.3 [15.1-111.9]	1.3 [0.6-2.7]	23.5 [7.5-63.4]	2.8 [1.1-6.0]	47.4 [15.0-120.2]	1.4 [0.6-3.0]	25.6 [7.7-69.8]	3.0 [1.1-6.7]
Rye bread	50.7 [18.1-112.7]	1.5 [0.8-2.5]	12.9 [4.6-28.9]	1.5 [0.8-2.4]	50.5 [17.3-114.9]	1.5 [0.7-2.5]	13.2 [4.6-29.8]	1.5 [0.7-2.5]
Red meat & processed meat	386.5 [153.6-778.1]	11.3 [7.5-15.2]	47.5 [18.3-98.2]	5.3 [3.4-7.5]	416.8 [170.1-816.7]	12.1 [8.2-16.0]	53.5 [21.4-106.1]	5.7 [3.8-8.0]
Fat spreads	53.1 [20.1-114.8]	1.5 [0.9-2.3]	10.1 [3.8-21.6]	1.1 [0.7-1.7]	56.3 [20.3-122.0]	1.6 [0.9-2.5]	10.9 [4.0-23.2]	1.2 [0.7-1.8]
Sweets & chocolate	119.2 [48.9 - 243.4]	3.5 [1.9-5.8]	10.3 [4.1-21.6]	1.2 [0.6-2.0]	109.9 [42.1-232.7]	3.2 [1.7-5.5]	9.5 [3.6-20.8]	1.1 [0.5-1.9]

^a^Purchases are aggregated over a 2-year period from January 1, 2017, to December 31, 2018 (€1=US $1.13 in 2017).

^b^Share of the food group purchase among all grocery purchases measured in euros.

^c^Share of the food group purchase among all grocery purchases measured in kilograms.

### Degree of Loyalty

Table 1 shows the self-assessed degree of loyalty. Almost 40% (14,194/36,526) of the participants reported that they made 80% or more of their food purchases at S Group shops and supermarkets, and 64% (23,418/36,526) reported making at least 60% of their purchases at the retailer’s shops and supermarkets.

The RFM scores were significantly different among the five degree of loyalty groups, with the lowest scores in the lowest degree of loyalty group and a steady increasing trend toward the highest degree of loyalty group (*F*_4_=4625.5, *P*<.001). The poststratification weights also differed significantly across the five groups (*F*_4_=24.1, *P*<.001), indicating that the degree of loyalty was associated with individuals’ sociodemographic characteristics. However, the observed differences were rather small, with a maximum difference of six percentage points between the groups (Table 3). In the highest degree of loyalty group, there were slightly more young and married participants, and the percentage of households with children was higher, whereas the percentage of divorced or separated participants and those with a master’s degree declined with the degree of loyalty.

**Table 3:** LoCard participant characteristics and RFM scores across the five degree of loyalty groups.

Characteristic			Degree of loyalty								
			0%-20% (n=2283)		21%-40% (n=4670)		41%-60% (n=6155)		61%-80% (n=9224)		81%-100% (n=14,194)
RFM^a^ analysis score, median [IQR]			182.5 [111.0-321.0]		311.0 [122.0-442.0]		335.0 [221.0-522.0]		432.0 [244.0-534.0]		445.0 [324.0-545.0]
Sex (women), n (%)			1472 (64.6%)		3176 (68.1%)		4150 (67.5)%		6101 (66.2)%		9317 (65.7%)
**Age, n (%)**											
	≤29	227 (10.0%)		571 (12.2%)		866 (14.1%)		1284 (13.9%)		2089 (14.7%)	
	30-39	406 (17.8%)		935 (20.0%)		1232 (20.0%)		1993 (21.6%)		3107 (21.9%)	
	40-49	512 (22.5%)		1003 (21.5%)		1327 (21.6%)		1838 (19.9%)		2779 (19.6%)	
	50-59	536 (23.5%)		1035 (22.2%)		1273 (20.7%)		1763 (19.1%)		2664 (18.8%)	
	60-69	411 (18%)		797 (17.1%)		1003 (16.3%)		1585 (17.2%)		2409 (17.0%)	
	≥70	188 (8.2%)		325 (7.0%)		450 (7.3%)		759 (8.2%)		1143 (8.1%)	
**Marital status, n (%)**											
	Presently married	1021 (45.0%)		2039 (43.9%)		2733 (44.5%)		4379 (47.6%)		7056 (49.8%)	
	Cohabitating	437 (19.3%)		982 (21.1%)		1321 (21.5%)		1862 (20.3%)		2803 (19.8%)	
	Single	416 (18.3%)		910 (19.6%)		1187 (19.3%)		1593 (17.3%)		2303 (16.3%)	
	Divorced or separated	323 (14.2%)		600 (12.9%)		729 (11.9%)		1090 (11.9%)		1588 (11.2%)	
	Widowed	73 (3.2%)		116 (2.5%)		166 (2.7%)		271 (3.0%)		412 (2.9%)	
**Household, n (%)**											
	Children aged under 18 years living in the household	649 (28.5%)		1398 (30.0%)		1846 (30.1%)		2995 (32.5%)		4814 (34.0%)	
**Education, n (%)**											
	Primary school or less	144 (6.3%)		231 (5.0%)		335 (5.5%)		525 (5.7%)		1023 (7.3%)	
	Upper secondary school	756 (33.3%)		1645 (35.4%)		2201 (36.0%)		3343 (36.4%)		5451 (38.6%)	
	Bachelor’s degree or equivalent	735 (32.4%)		1538 (33.1%)		2046 (33.4%)		3020 (32.9%)		4446 (31.5%)	
	Master’s degree or higher	635 (28.0%)		1228 (26.5%)		1538 (25.1%)		2302 (25.1%)		3189 (22.6%)	
**Occupational status, n (%)**											
	Employed	1348 (59.2%)		2892 (62.2%)		3761 (61.2%)		5544 (60.2%)		8533 (60.2%)	
	Unemployed	124 (5.5%)		219 (4.7%)		284 (4.6%)		408 (4.4%)		602 (4.3%)	
	Student	125 (5.5%)		236 (5.1%)		329 (5.4%)		461 (5.0%)		673 (4.7%)	
	Retired	563 (24.7%)		1018 (21.9%)		1381 (22.5%)		2207 (24.0%)		3391 (23.9%)	
	Parental leave	51 (2.2%)		137 (2.9%)		174 (2.8%)		318 (3.5%)		575 (4.1%)	
	Other	65 (2.9%)		151 (3.2%)		214 (3.5%)		273 (3.0%)		402 (2.8%)	

^a^RFM: recency, frequency, and monetary.

Table 4 shows food purchases in the degree of loyalty groups, and all showed significant associations (*P*<.001 for all food groups, except sweets & chocolate [*P*=.007]). The result was expected owing to the large sample size. The shares of vegetable, red meat & processed meat, and fat spread purchases increased as the degree of loyalty increased. In the other food groups, there were no major differences across the degree of loyalty groups.

Additionally, Table 4 shows that the quantity and expenditure regarding food groups increased steadily with the self-assessed degree of loyalty, suggesting that the self-assessment can be relied upon.

**Table 4:** Purchases (in € and kg) of selected food groups across the five degree of loyalty groups.

Food group	Degree of loyalty																			
	0%-20% (n=2216)				21%-40% (n=4611)				41%-60% (n=6119)				61%-80% (n=9168)				81%-100% (n=14,133)			
	€^a,b^	€%^b,c^	kg^b^	kg%^b,d^	€^b^	€%^b,c^	kg^b^	kg%^b,d^	€^b^	€%^b,c^	kg^b^	kg%^b,d^	€^b^	€%^b,c^	kg^b^	kg%^b,d^	€^b^	€%^b,c^	kg^b^	kg%^b,d^
Vegetables	58.0	6.6	15.2	7.1	131.1	7.4	35.0	7.9	232.0	7.9	60.6	8.4	344.2	8.1	93.7	8.7	441.9	7.9	122.5	8.5
Skimmed milk & sour milk	1.8	0.2	2.0	0.8	3.6	0.2	3.0	0.9	5.6	0.2	5.0	0.8	8.8	0.2	8.0	0.9	14.0	0.3	13.5	1.1
Sugar-sweetened beverages	10.9	1.3	5.5	2.7	23.7	1.4	12.2	2.9	37.2	1.3	19.1	2.8	52.2	1.3	27.4	2.7	67.9	1.3	35.7	2.7
Rye bread	10.9	1.3	2.7	1.3	24.1	1.4	6.2	1.4	41.9	1.5	10.4	1.5	61.9	1.5	15.5	1.5	82.9	1.5	21.4	1.5
Red meat & processed meat	80.4	9.9	9.9	5.0	186.4	10.7	22.8	5.2	297.8	10.9	36.8	5.2	457.4	11.3	55.2	5.3	621.5	11.6	76.9	5.4
Fat spreads	10.2	1.2	1.8	0.9	23.5	1.3	4.4	1.0	41.0	1.4	7.8	1.1	65.4	1.6	12.3	1.2	87.2	1.6	16.7	1.2
Sweets & chocolate	32.3	3.7	2.8	1.3	64.1	3.7	5.4	1.3	95.4	3.4	8.3	1.2	134.6	3.3	11.6	1.1	183.9	3.5	15.6	1.2
Total amount of grocery purchases, median [IQR]	873.4 [442.0-1602.5]		215.9 [105.6-393.2]		1883.8 [1095.7-2924.2]		460.7 [258.5-756.1]		2958.6 [1840.0-4528.8]		734.0 [452.8-1169.5]		4320.2 [2726.5-6479.0]		1095.3 [671.5-1698.1]		5680.1 [3616.4-8531.8]		1462.7 [913.0-2250.6]	

^a^€1=US $1.13 in 2017.

^b^Median value.

^c^Share of the food group purchase among all grocery purchases measured in euros.

^d^Share of the food group purchase among all grocery purchases measured in kilograms.

## Discussion

### Principal Findings

The findings of this study showed that individuals who consented to the release of their loyalty card data for research purposes tended to diverge from the general Finnish adult population. Similar to many other health and nutrition studies, including those encompassing electronic data collection tools [7,13,35,40,41], the LoCard participants manifested volunteer bias, with employed individuals, middle-aged individuals, women, and individuals with higher education being overrepresented in the sample. The LoCard sample included fewer retired individuals, fewer individuals with basic education, and fewer individuals who had children aged under 18 years living in the household. Compared with the Finnish national FinHealth and FinDiet studies, the selection mechanism appeared to be somewhat different in the LoCard sample. While employed individuals were overrepresented in all these three studies, the gender and education biases were stronger in the LoCard sample. Moreover, the LoCard sample had a rather similar distribution of marital status as among Finnish adults, whereas in the FinHealth study, married individuals were overrepresented [35,36,42].

However, the size (n=47,066) and heterogeneity of the LoCard sample enabled a successful correction of the differences seen in the sociodemographic variables. We developed the poststratification weights using all sociodemographic background variables available with the two-way joint distributions to correct the background distributions of the LoCard participants to make them closer to the Finnish adult population. The large sample size provided a sufficient number of participants for hard-to-reach population subgroups, and thus, it was possible to construct the poststratification weights for them as well. The highest weights were seen for unmarried men aged under 30 years, who indeed are often underrepresented or not enrolled in health studies [41].

Of the 1.1 million loyalty card holders contacted, approximately 4% (n=47,066) took part in the LoCard study. Although low, the participation rate was similar to that for other massive data collection methods [7]. The advantage of the use of digital tools is that they reach a large number of potential study participants with relatively low effort in data collection. After all, we reached substantially more individuals than in the majority of dietary studies using traditional data collection methods with minimum human involvement in data collection. A likely reason for the low participation rate was that the participants were contacted via email, which may not have reached them (invalid email address or contact email classified as “junk email”) or may have limited their participation and induced selection bias. Although 88% of households in Finland have an internet connection and 83% use email [43], email use varies according to sociodemographic profiles and is relatively low at 62% among individuals aged over 65 years and among individuals with basic education [44]. This may partly explain the baseline characteristics of the LoCard sample. However, it has been shown that the use of digital tools in recruitment and data collection does not increase the selection bias, but the traits of participants in health studies are rather similar regardless of the recruitment method used [13,40]. Moreover, it is likely that many simply ignored an email coming from a commercial party.

Important aspects are whether and when informed consent from loyalty card owners is needed. Recently, Aiello et al [45] published an interesting ecological study on the associations between loyalty card food purchase data and prescription records that were used as a proxy for real disease profiles in London. Their dataset included 1.6 million loyalty card users, and they used the anonymized data without the consent of the individuals. In our study, consent and a positive reply were required for two reasons. First, transparent use of loyalty card data on customers for a common good builds trust among them, researchers, and the company, and reduces the likelihood of negative publicity. Second, contact was needed to obtain information about participants’ background characteristics for use in further analyses. A future scenario could involve a consent request when the customer becomes a member of the loyalty card program. This would create an ethically sound and transparent research protocol for the use of customer data.

Poststratification weights were further applied in evaluating the purchases of the main food groups. The corrections demonstrated small changes in some food groups; the purchase of vegetables and sweets & chocolate decreased after the correction, whereas the purchase of red meat & processed meat, sugar-sweetened beverages, and fat spreads increased*.* The sociodemographic profiles of the LoCard participants and bias related to them might, at least partly, explain these results. The FinDiet study showed that women, who were overrepresented in the LoCard sample and thus had smaller weights, tended to consume more fruits and vegetables than men [36]. In line with this, after applying the poststratification weights, the purchase of vegetables decreased. It has also been shown that socioeconomically advantaged individuals, who likewise were overrepresented in the LoCard sample, consumed healthy foods, such as fruits and vegetables and low-fat dairy products, more frequently [46]. Moreover, the increased amount of red meat & processed meat purchase is likely related to male participants who tend to consume more meat [36,47]. In line with the overall findings of this study, the NutriNet-Santé study showed that the consumption of fruits and vegetables was higher and the consumption of meat was lower in the cohort than in the general population in France [13].

The degree of loyalty was fairly high in the LoCard sample with 64% (23,418/36,526) of the participants reporting making over 60% of their grocery purchases at the retailer’s shops and supermarkets. The food purchases were rather similar in the higher loyalty group (60% or higher), whereas individuals making less purchases in the retailer’s grocery stores showed some differences. In particular, individuals reporting the lowest degree of loyalty tended to buy fewer vegetables and fat spreads and fewer red meat & processed meat products. Although some variation was seen, the differences across the loyalty groups were smaller than expected. One reason could be the food groups selected for the current analyses. There could be other products, such as alcohol and tobacco, that are differently purchased. These results, together with the differences seen in the sociodemographic variables between the loyalty groups, underline the importance of estimating and accounting for the degree of loyalty in future studies using loyalty card data. A direct way to address the problem of coincidental purchases is to focus on a subsample with at least 60% loyalty. It is also important to note that loyalty card data can enable research on longitudinal trends in food purchases, which can be performed regardless of the degree of loyalty.

### Limitations

Although we used a large set of matching variables for developing the poststratification weights, some limitations concerning these remain. First, we were not able to compare or account for possible differences in income, as there was no comparable reference data available in Statistics Finland. Therefore, it remains unclear whether the LoCard sample was representative in terms of income, which is an important contributor to food purchase. The higher education level of the LoCard participants and the lower prevalence of young and retired individuals clearly suggest that the income levels might be overestimated in our sample. Second, although we matched families with children, the number of children and their ages, which can clearly affect a household’s food purchases, were not used in weighting. Importantly, we were able to correct the differences only in the observed sociodemographic variables, and thus, unidentifiable selection bias cannot be ruled out. This may include factors that would be associated with willingness to participate, such as special dietary restrictions and socially excluded people. In particular, among those participants who did not have complete background information and whose poststratification weights were thus based on sex and age only, the risk for unidentifiable selection bias could be even higher.

It is important to note that grocery purchases reflect consumption on a household level, which may consist of more than one person, and not everybody might eat the same foods. Thus, accuracy of loyalty card data in investigating individual diet may not be as high as that obtained with traditional data collection methods. However, good compatibility between respondent-collected household-level food purchase data and individual-level dietary data has been demonstrated [28,48]. Moreover, foods purchased from stores do not necessarily indicate foods consumed owing to many different reasons. These include, for example, foods that are not included in loyalty card data, such as dinner foods at restaurants or lunch foods at work. Cardholders may also buy foods that are consumed by others, for example, grandchildren, other relatives or close friends invited for dinner, and pets. Some foods are not consumed at all, resulting in food wastage [49,50].

Finally, the degree of loyalty remains a challenge. In this study, the degree of loyalty was self-estimated, and it is difficult to estimate possible bias related to this self-report. However, we showed that the RFM scores increased steadily with the groups of loyalty, indicating that higher frequency, higher engagement, and more money spent on grocery purchases were associated with a higher degree of loyalty. Moreover, a positive correlation was found between the proportional increases in money spent (€) and weight (kg) regarding food purchases and the degree of loyalty. These results suggest that this self-assessment seemed to provide a feasible estimate of the true values. In another study, the researchers defined loyalty through the frequency of purchases made in the supermarket combined with the amount of money spent on purchases. However, in this study, the degree of loyalty was not specifically defined [51].

Despite its limitations, we see real potential in the use of these automatically collected longitudinal food purchase data in the population-based assessment of dietary patterns, which are important determinants of health and carbon footprint [52]. Loyalty card data provide a cost-effective tool to reach large groups of individuals with minimum data collection efforts and to investigate diet-related behaviors with less information bias. Linking these data with other health data (such as electronic health records and health registers) would provide new opportunities to understand diet and related outcomes. However, such research settings include privacy concerns that need to be carefully addressed to guarantee individual anonymity and consent. In addition, loyalty card data enable the monitoring of longitudinal trends in food purchases including timely monitoring and evaluation of the impact of population-level steering instruments such as taxation.

### Conclusions

Individuals who consented to the use of their loyalty card data for research purposes tended to differ from individuals in the general Finnish adult population. The sociodemographic distributions were toward similar characteristics, as is frequently seen in health and nutrition studies. However, the high volume of data enabled the inclusion of sociodemographically heterogeneous subgroups, potentially including hard-to-reach subgroups, and further correction of the differences so that distributions matched well with those of the general Finnish adult population. A potential confounder in studies using loyalty card data is the degree of loyalty, which in this study, was associated with food-purchasing profiles and also the participants’ background characteristics. This underlines the importance of obtaining sufficient background information when using loyalty card data for health research.

Despite the limitations, loyalty card data provide a cost-effective approach for large groups of individuals with minimum data collection effort and for the investigation of diet-related behaviors on a large scale with less information bias. Importantly, loyalty card data enable the monitoring of longitudinal trends in grocery purchases.

## Appendix

Multimedia Appendix 1 Grouping of the food purchase data.

Multimedia Appendix 2 The percentage of individuals living in each of the 19 regions in Finland, in the LoCard sample, and in the weighted LoCard sample.

Multimedia Appendix 3 Regions of Finland.

## Abbreviations

IPF: iterative proportional fitting
RFM: recency, frequency, and monetary
