# Supplementary material for: Characterization and Correction of Bias Due to Nonparticipation and the Degree of Loyalty in Large-Scale Finnish Loyalty Card Data on Grocery Purchases: Cohort Study
Source: J Med Internet Res. 2020 Jul 15;22(7):e18059. doi: 10.2196/18059 (PMC7392131; doi:10.2196/18059)
Supplement: Multimedia Appendix 1 [file jmir_v22i7e18059_app1.docx]

Grouping of the food purchase data

The food expenditure data received from the S group consisted of 4, 234 different product groups that were categorized into six product category levels. The data were not grouped into clearly demarcated functional categories for the purposes of studies on diet and health: for instance, new plant-based protein products were classified into the same category as whole meat products. Therefore, it was necessary to do re-grouping.

**I phase** We identified food groups from the grocery product groups. This was performed on the crudest product category level including altogether 27 product groups. For example, “Fruit and vegetables” or “Fresh meat” were considered food groups but “Tobacco” or “Hygiene” were left out.

**II phase** We re-grouped all the food groups on the basis of the commonly used food groupings in nutritional studies and in earlier findings on the associations between dietary components and health. We aimed to form groups that could be used as indicators of the nutritional quality of the household food purchases. The re-grouping was primarily done according to the name of the food group. For instance, “Cucumbers” were re-grouped into “Vegetables”. In many cases the name of the food group was undefinable and further investigation was needed. “Other grilled products” is an example of an unclearly named food group. The content of the group was then examined via retailer’s webpage www.foodie.fi or product list provided by S group. After thorough investigation, some food groups remained unclassified due to lack of product information or being a mixed dish with no definite primary ingredient. In total, 42 food groups were left out due to being either i) a mixed dish or food group with no definite primary ingredient, or ii) a rarely purchased product.

Out of the 4 234 grocery product groups, 865 (20%) were assigned into one of the new food groups used in

the present study.

Examples of the food products included in the new food groups:

*Vegetables*: fresh, canned, and frozen vegetables

*Skimmed milk & sour milk*: regular, low-lactose and lactose-free skimmed milk, and skimmed sour milk

*Sugar-sweetened beverages*: soft drinks, energy drinks, juices, ice teas, and seasonal drinks

*Rye bread*: fresh and crisp rye bread, ryemeal bread

*Red meat & processed meat*: Rred meat, cold cuts, meat dishes, sausages, processed red and white meat

*Fat spreads*: margarine, butter, and fat blends

*Sweets & chocolate*: sweets, chocolate, and coated nuts

A complete list of used food groups is available on request.
