# Supplementary material for: Characterization and Correction of Bias Due to Nonparticipation and the Degree of Loyalty in Large-Scale Finnish Loyalty Card Data on Grocery Purchases: Cohort Study
Source: J Med Internet Res. 2020 Jul 15;22(7):e18059. doi: 10.2196/18059 (PMC7392131; doi:10.2196/18059)
Supplement: Multimedia Appendix 2 [file jmir_v22i7e18059_app2.docx]

|  | **Finnish Population** | **LoCard sample** |  | **Weighted LoCard sample** |
| --- | --- | --- | --- | --- |
| Lapland [%] | 3 | 2 |  | 2 |
| North Ostrobothnia [%] | 8 | 5 |  | 5 |
| Kainuu [%] | 1 | 1 |  | 1 |
| North Karelia [%] | 3 | 3 |  | 3 |
| North Savo [%] | 5 | 5 |  | 5 |
| South Savo [%] | 3 | 2 |  | 3 |
| South Karelia [%] | 2 | 2 |  | 2 |
| Central Finland [%] | 5 | 6 |  | 6 |
| South Ostrobothnia [%] | 4 | 2 |  | 3 |
| Ostrobothnia [%] | 3 | 2 |  | 2 |
| Central Ostrobothnia [%] | 1 | 1 |  | 1 |
| Pirkanmaa [%] | 9 | 11 |  | 11 |
| Satakunta [%] | 4 | 3 |  | 4 |
| Päijät-Häme [%] | 4 | 4 |  | 4 |
| Kanta-Häme [%] | 3 | 4 |  | 4 |
| Kymenlaakso [%] | 3 | 3 |  | 3 |
| Uusimaa [%] | 30 | 37 |  | 35 |
| Southwest Finland [%] | 9 | 8 |  | 8 |
| Åland [%] | < 1% | < 1% |  | < 1% |

Table S1. The percentage of individuals living in each of the 19 regions in Finland, in the LoCard sample, and in the weighted LoCard sample
