# Supplementary figures and images for: Characterization and Correction of Bias Due to Nonparticipation and the Degree of Loyalty in Large-Scale Finnish Loyalty Card Data on Grocery Purchases: Cohort Study
Source: J Med Internet Res. 2020 Jul 15;22(7):e18059. doi: 10.2196/18059 (PMC7392131; doi:10.2196/18059)

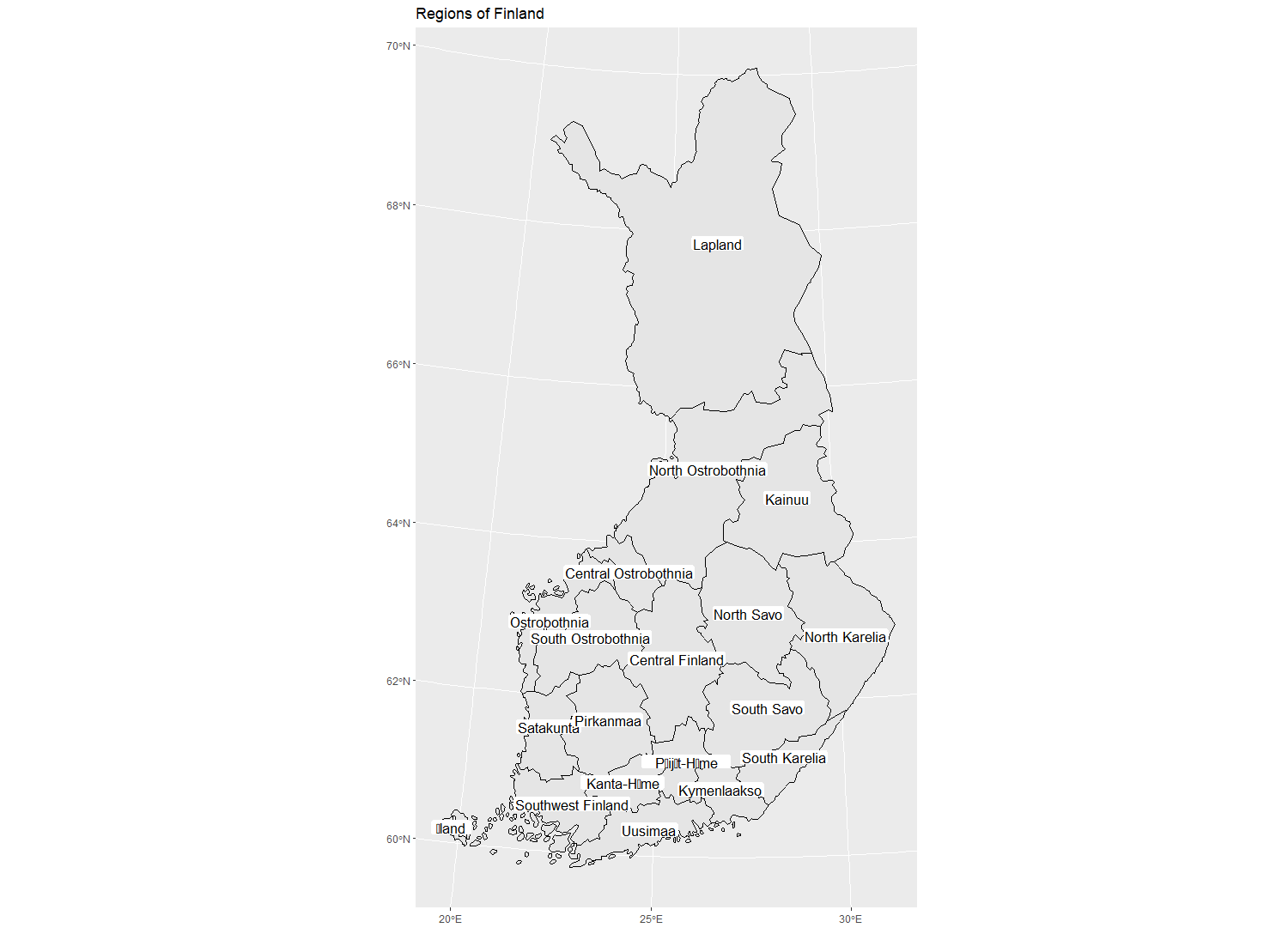

Supplement: Multimedia Appendix 3 [file jmir_v22i7e18059_app3.png]
